# Overweight, the Cardiovascular Risk of the Century

**DOI:** 10.5935/abc.20190171

**Published:** 2019-08

**Authors:** Paulo César B. Veiga Jardim

**Affiliations:** Faculdade de Medicina da Universidade Federal de Goiás, Goiânia, GO - Brazil; Liga de Hipertensão Arterial da Universidade Federal de Goiás, Goiânia, GO - Brazil; Hospital do Coração de Goiás, Goiânia, GO - Brazil

**Keywords:** Cardiovascular Diseases, Risk Factors, Prevention & Control, Overweight, Indicators of Morbidity and Mortality, Feeding Behavior/trends, Obesity

Over time, the scientific community has accurately identified the main cardiovascular risk factors. There is no doubt about their importance, the weight of each one in determining the increase in morbidity and mortality due to this cause.^1^

On the other hand, the temporal growth of circulatory system pathologies as the main cause of death and leave from work was gigantic. An untold social, economic and affective harm.

Scientific development has enabled the emergence of instruments and drugs to address major modifiable risk factors.

Ironically though, is that, in general, from the point of view of disease control, we make more progress in secondary prevention than in primary prevention.^1^

In secondary prevention, combating some of the greatest risk factors has greater appeal. Treatment of hypertension, dyslipidemia and even diabetes has evolved a lot. Platelet antiaggregation and anticoagulation as a means of preventing further events have also gone a long way. In this case, despite the problems related to lack of access or adherence to treatment, we have cleared the ground and keep on moving.^1^

Regarding lifestyle habits, there are multiple answers even in this situation. Smoking cessation is well accepted and, due to the actions taken on all levels in our country, the results are very encouraging. However, there is greater resistance to changing sedentary behaviors, despite the dissemination of knowledge about its importance. Psychosocial stress is another factor that needs further studies and more effective actions.^1^

However, there is a major public health challenge against which we have been sustaining setbacks year after year.

## The challenge of the century: overweight

Published studies have shown that, worldwide, over the past 50 years, the population has increased in weight. A 2014 publication reported that between 1980 and 2013 individuals had an increase in body mass index above 25 kg/m^2^, which classifies them as overweight, from 28.8% to 36.9% among men and 29.8% to 38.0% among women. Worse than that, children and adolescents also had weight gain both in developed countries where, in 2013, 23.8% of boys and 22.6% of girls were overweight or obese, and in developing countries, where 12.9% of boys and 13.4% of girls were also overweight.^2^

In 2016, another publication that evaluated the period from 1975 to 2014 also showed that obesity increased from 3.2% to 10.8% among men and from 6.4% to 14.9% among women. These studies estimate that if this trend continues, in 2025, the prevalence of obesity around the world will be greater than 18% among men and 21% among women.^3^

This said, we clearly have a worldwide overweight pandemic, made worse by the fact that there has not been, so far, any description of any developed program that has succeeded in stopping this harsh reality.

This is an important cardiovascular risk that went unnoticed initially, which is taking alarming proportions and gaining momentum.

It should be noted that, in 2015, overweight drove more than 100 million people away from their jobs and was responsible for about 4 million deaths worldwide.^4^

In Brazil it is no different: the epidemic is severe and progresses noticeably. From 2006 to 2016, in a survey using VIGITEL data, which somewhat underestimates information, the prevalence of overweight increased from 48.1% to 57.5% among men and from 37.8% to 48.2% among women, and obesity increased from 11.7% to 18.1% among men and from 12.1% to 18.8% among women.^5^

Another major longitudinal study - ELSA-Brasil - showed in a 2015 publication, in a population aged 35 to 74, a prevalence of 40.2% of overweight individuals and 22.9% of obese individuals.^6^ It is scary, but there is more.

These surveys report data from capital cities and/or large urban centers and, when we seek information about small towns, we find the same reality.

**Figure 1:**
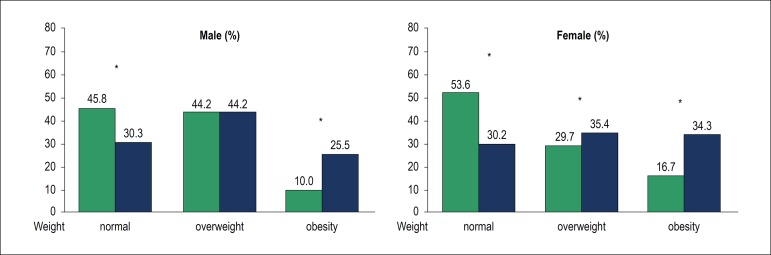
Nutritional status based on body mass index (n = 685). Firminópolis, Brazil (2002-2015). *Significant p = 0.05.

For example, a 13-year longitudinal study in a small town in the Midwest of Brazil, in a population of people older than 18, found an increase in overweight/obesity, which was already high in 2002, from 49.1% to 69.8% in 2015. As atypical as it may seem and even more challenging, overweight in the period increased from 34.6% to 38.4%, while obesity increased from 14.5% to a scary rate of 31.4%. Note that the same individuals were investigated at two different times. Considering stratification by gender, there was a decrease in the number of normal weight individuals and an increase in obesity in that time frame.^7^ (Figure 1)

It is also worth noting that in Brazil, even in children, from very young kids to teenagers, there are impressive percentages of overweight and obesity.

A study with children aged 2 to 5 in midwestern Brazil found 11.2% of overweight.^8^ Another sample of 3169 slightly older school children (7 to 14 years old), also in the Midwest region, found 16% of overweight children and 4.9% of obese children, revealing the same trend since childhood.^9^

Finally, wrapping up the cycle, in the evaluation of school adolescents (12 to 17 years old), two population-based studies, one representing a city and another representing the whole country - the studies CORADO and ERICA - found overweight percentages of 23.3% and 17.1%, respectively.^10,11^

There is no other way of looking at it: it is an epidemic, it ravages the world, it grows rapidly and is not effectively tackled.

The scientific community has not realized the seriousness of this issue, it still works from a “treatment” perspective and is very shy when it comes to primary prevention, as it was clearly outlined in recent documents from the European Society of Hypertension and the European Association for the Study of obesity.^12,13^

We already have strong indications that incentives or even restrictive measures with taxation of certain products that may be considered harmful are cost-effective and may potentially lead us to a safer spot.^14,15^

It is really a time for taking action, for us to stop being doctors of illness and acting from the perspective of real healthcare professionals. We should all make more of an effort. And that includes each individual from society and especially from the government.

Tackling overweight should be a government policy in pursuit of an effective action nationwide, otherwise we will move towards an even darker future in terms of cardiovascular disease.
